# Macrophage Inhibitory Cytokine-1 (MIC-1/GDF15) Slows Cancer Development but Increases Metastases in TRAMP Prostate Cancer Prone Mice

**DOI:** 10.1371/journal.pone.0043833

**Published:** 2012-08-27

**Authors:** Yasmin Husaini, Min Ru Qiu, Glen P. Lockwood, Xu Wei Luo, Ping Shang, Tamara Kuffner, Vicky Wang-Wei Tsai, Lele Jiang, Pamela J. Russell, David A. Brown, Samuel N. Breit

**Affiliations:** 1 St Vincent’s Centre for Applied Medical Research, St Vincent’s Hospital and University of New South Wales, Sydney, New South Wales, Australia; 2 Department of Anatomical Pathology, SydPath, St Vincent’s Hospital, Sydney, New South Wales, Australia; 3 Australian Prostate Cancer Research Centre-Queensland, Institute of Health and Biomedical Innovation, Queensland University of Technology, Princess Alexandra Hospital, Brisbane, Queensland, Australia; Queensland University of Technology, Australia

## Abstract

Macrophage inhibitory cytokine-1 (MIC-1/GDF15), a divergent member of the TGF-β superfamily, is over-expressed by many common cancers including those of the prostate (PCa) and its expression is linked to cancer outcome. We have evaluated the effect of MIC-1/GDF15 overexpression on PCa development and spread in the TRAMP transgenic model of spontaneous prostate cancer. TRAMP mice were crossed with MIC-1/GDF15 overexpressing mice (MIC-1^fms^) to produce syngeneic TRAMP^fmsmic-1^ mice. Survival rate, prostate tumor size, histopathological grades and extent of distant organ metastases were compared. Metastasis of TC1-T5, an androgen independent TRAMP cell line that lacks MIC-1/GDF15 expression, was compared by injecting intravenously into MIC-1^fms^ and syngeneic C57BL/6 mice. Whilst TRAMP^fmsmic-1^ survived on average 7.4 weeks longer, had significantly smaller genitourinary (GU) tumors and lower PCa histopathological grades than TRAMP mice, more of these mice developed distant organ metastases. Additionally, a higher number of TC1-T5 lung tumor colonies were observed in MIC-1^fms^ mice than syngeneic WT C57BL/6 mice. Our studies strongly suggest that MIC-1/GDF15 has complex actions on tumor behavior: it limits local tumor growth but may with advancing disease, promote metastases. As MIC-1/GDF15 is induced by all cancer treatments and metastasis is the major cause of cancer treatment failure and cancer deaths, these results, if applicable to humans, may have a direct impact on patient care.

## Introduction

Prostate cancer (PCa) is the most commonly diagnosed cancer in men after skin cancer and is the second leading cause of male cancer deaths. In 2011, in the USA alone, there were 240,890 new cases of PCa and approximately 33,720 deaths [1]. PCa, like many cancers, is characterized by the altered expression of cytokines and growth factors. One such cytokine is MIC-1/GDF15, a divergent member of the transforming growth factor-β (TGF-β) superfamily [2] that is over-expressed by many patients with common cancers including those of the prostate and can be further induced by cancer therapies including surgery, chemo and radio-therapy of prostate, colon and breast cancer [3]–[9].

MIC-1/GDF15 protein expression is markedly enhanced in cancer tissues, and cancer cell lines. In cancers, such as that of the prostate, the major source of MIC-1/GDF15 is the malignant epithelial cell itself [5], [10], although there may also be a contribution from tumor stromal cells [11] and infiltrating phagocytes [12]. This tumor expression of MIC-1/GDF15 is often reflected in its blood levels, which increase with cancer development and progression [5], [10], [13]–[27], generally in proportion to the stage and extent of disease. For example, serum levels rise progressively in patients from normal, to low-grade colonic polyps, high-grade colonic polyps, localized colon cancer and finally disseminated colon cancer [13]. Patients with colon cancer who have elevated serum MIC-1/GDF15 levels have a worse overall prognosis and earlier disease relapse [13], [25]. Similar results have been observed for many other malignancies including melanoma [28], [29] and cancers of the pancreas [12], [19], [30], thyroid [31], [32], ovary [26] and endometrium [27]. In prostate cancer patients, serum MIC-1/GDF15 concentrations independently predict bone metastasis and overall survival [14], [21].

In patients with advanced cancers, serum MIC-1/GDF15 levels rise from an average in non-cancer patients of about 450 pg/ml [13] to up to 10,000–100,000 pg/ml [5]. Because of its effects on feeding centres within the brain, elevation in serum levels of MIC-1/GDF15 is an important cause of cancer-associated anorexia/cachexia [33]. The amount of MIC-1/GDF15 present in serum of cancer patients is, in part, dependent on the proportion of MIC-1/GDF15 localized to the tumor versus that diffusing into the circulation [34]. The variable processing of MIC-1/GDF15 results in secretion in both an unprocessed (with propeptide) and a processed (without propeptide) form [34]. As its propeptide domain binds to extracellular matrix, unprocessed MIC-1/GDF15 remains localized to the tumor stroma, whilst the processed form rapidly diffuses into the circulation [12], [34]. In PCa, an increased amount of MIC-1/GDF15 localized to the tumor improves patient outcome, especially in those with a Gleason sum score of 6 or less [34].

Although there are multiple lines of evidence linking MIC-1/GDF15 to cancer in general and PCa specifically, convincing and consistent data on its biological role in the pathogenesis and progression of cancer is limited [4]. Evidence, largely from *in vitro* and xenograft experiments provide apparently contradictory results. The majority of studies have suggested that MIC-1/GDF15 has anticancer activity and induces tumor cell apoptosis [35]–[38]. For example, MIC-1/GDF15 transfected HCT-116, human colorectal cancer cells exhibited increased basal apoptosis, increased response to non-steroid anti-inflammatory drugs and reduced soft agar cloning efficiency [35]. The DU145 PCa cell line, one of the few that does not express MIC-1/GDF15, undergoes apoptosis when treated *in vitro* with MIC-1/GDF15 [36].

Contradicting the above data, there is also evidence from *in vitro* experiments that MIC-1/GDF15 may facilitate tumor progression [39]–[42]. For example, enforced MIC-1/GDF15 overexpression or treatment with purified recombinant MIC-1/GDF15 significantly increased the *in vitro* invasion of gastric cancer cell lines. This was mediated by an increase in urokinase type-plasminogen-activator (uPA) and involved activation of extracellular signal regulated kinase-1/2 (ERK-1/2) [16]. Overexpression and knockdown experiments in androgen-dependent LNCaP-C33 human PCa cell line and its highly metastatic variant, androgen-independent LNCaP-LN3 have shown that MIC-1/GDF15 can promote the proliferation of androgen receptor (AR^+^)-positive LNCaP cells via the stimulation of the ERK-1/2 signal pathway [20]. By contrast, others have not been able to identify any action of MIC-1/GDF15 on the *in vitro* proliferation of LNCaP cells [36].

The *in vivo* cancer related activity of MIC-1/GDF15, has also been examined in a limited number of tumor xenograft studies. Enforced MIC-1/GDF15 overexpression in HCT-116 colon cancer cells, xenografted into nude mice, resulted in reduction in tumor size [35]. A glioblastoma cell line, with no antiproliferative response to MIC-1/GDF15 *in vitro*, failed to grow as a tumor xenograft in nude mice when transfected with MIC-1/GDF15 [43]. In contrast, siRNA knock down of MIC-1/GDF15 in a melanoma cell line significantly reduced the growth of xenografted tumors [28].

Because MIC-1/GDF15 is so frequently and substantially overexpressed in cancers, and because anti-cancer treatments induce MIC-1/GDF15 expression both in cancer and non-cancer tissues, any effects on tumor behavior may be of high clinical significance. In order to obtain the most reliable data on the overall role of MIC-1/GDF15 in the biology of PCa, and by analogy cancer in general, we utilised *T*ransgenic *A*denocarcinoma of *M*ouse *P*rostate (TRAMP) prostate cancer prone mice. TRAMP mice express SV40 early genes (T and t; Tag) in the prostatic epithelium under the control of rat probasin (rPB) promoter [44]. Heterozygous TRAMP male mice develop progressive prostate cancer exhibiting the spectrum of disease as it occurs in men that is invasive and capable of metastatic spread to distant sites, primarily the pelvic lymph nodes, liver, kidney and lungs [45]. We crossed TRAMP mice with MIC-1/GDF15 overexpressing mice (MIC-1^fms^) to produce TRAMP mice that overexpress MIC-1/GDF15 (TRAMP^fmsmic-1^). In MIC-1^fms^ mice expression of MIC-1/GDF15 is from myeloid cells (macrophages, dendritic cells and neutrophils), and is sufficient to increase its serum levels by about 10–90 fold [33]. We have compared survival rate, pattern of PCa growth and spread in TRAMP and TRAMP^fmsmic-1^ mice. These studies demonstrate that whilst TRAMP^fmsmic-1^ mice have more slowly growing tumors, survive about 1/3 longer and have lower grade tumors, they develop much more metastases. Therefore, like TGF-β, MIC-1/GDF15 has a complex effect on tumor behavior, inhibiting local tumor growth, but enhancing metastatic spread.

## Materials and Methods

### Ethics Statement

All research and animal care procedures were approved by the Garvan Institute/St Vincent’s Hospital Animal Experimentation Ethics Committee (Ethics No: 07/05 and 10/05) and were in agreement with the Australian Code of Practice for the Care and Use of Animals for Scientific Purpose.

### Transgenic Mice

Male TRAMP mice heterozygous for the PB-Tag transgene, were generated by mating TRAMP^+/−^ females (C57BL/6 background) with non-transgenic C57BL/6 males. In TRAMP mice, the minimal rat probasin (rPB) regulatory sequence targets SV40 early gene (T and t; Tag) expression specifically to prostatic epithelium [44]. Syngeneic mice overexpressing MIC-1/GDF15 under control of the myeloid cell specific c-fms promoter (MIC-1^fms^) were used to breed TRAMP mice that also overexpress MIC-1/GDF15 (TRAMP^fmsmic-1^). MIC-1/GDF15 expression in MIC-1^fms^ mice is from myeloid cells, but is sufficient to also increase its serum levels by about 10–90 fold [33].

The double transgenic TRAMP^fmsmic-1^ mice were generated by crossing TRAMP^+/−^ females with homozygous MIC-1^fms^ males. The PB-SV40 T transgene was identified using DNA extracted from tail samples and PCR primers directed at the PB-SV40 T antigen sequence: Pb-forward: 5′-CCGGTCGACCGGAAGCTTCCACAAGTGCATTTA-3′ and SV40Tag-reverse: 5′-CTCCTTTCAAGACCTAGAAGGTCCA-3′. PCR was performed using reaction mixture and conditions described previously [46]. The MIC-1/GDF15 transgene in TRAMP^fmsmic-1^ mice was identified by PCR using primers, Flag-forward: 5′-GACTACAAGGACGACGATGACAAG-3′ and MS8-reverse: 5′-CGAAGCCTACCGCGTGCACCGAG-3′ and reaction conditions: denaturation at 95°C for 10 s, annealing at 60°C for 20 s, and extension at 72°C for 30 s.

### Survival Study

Based on the statistical power analysis for sample size, 35 TRAMP and 35 TRAMP^fmsmic-1^ mice were allocated at 4–6 weeks of age, for a survival study. From that time, mice were weighed once a week and monitored twice a week for tumor size and extent by palpating the abdomen. Mice either died or were culled when they reached ethical end points of tumor size larger than 11 mm×11 mm×11 mm, more than 20% weight loss or meeting any other ethical end point criteria for euthanasia. The overall survival of individual mice was calculated from birth to ethical end point or death from tumor. Survival distribution was estimated using the method of Kaplan-Meier. At necropsy the genitourinary complex (GU) consisting of prostate (including dorsal, lateral, ventral, and anterior lobes), urethra, ampullary gland, seminal vesicle (SV) and urinary bladder was taken out and weighed. Weight of the GU, prostate, and SV of each mouse was normalized by its body weight (organ wt/body wt).

### Primary Tumor Size

In a separate cohort to that above, GU and prostate tumor growth was compared in TRAMP and TRAMP^fmsmic-1^ mice. At the start of the study 60 TRAMP and 60 TRAMP^fmsmic-1^ mice, 15 of each for each stage, were pre-allocated to be sacrificed at different time points from early to advanced tumor stages (8, 17, 25 and 33 weeks of age). An additional 3 mice were assigned to the TRAMP, 33-week group as 3 of the original mice in this group died or reached ethical end points between 23 and 33 weeks of age. For each of the 60 mice necropsied, the GU was excised. Total GU, prostate and seminal vesicle weight were measured and prostatic dimensions recorded. All measurements were normalized for the donor mouse total body weight. After this, the tissue was used for prostate tumor grading.

### Prostate Tumor Grading

Prostate tissues from 8, 17, 25 and 33 weeks old TRAMP and TRAMP^fmsmic-1^ mice above, were placed in a cassette and fixed in 10% neutral buffered formalin. Care was taken to place and embed prostates from all the mice in this experiment in the same orientation. Sections were taken at 200*-*µm intervals until the tissue in the block was exhausted. Slides were stained with haematoxylin and eosin and all the sections for individual animals were examined using light microscopy, by a histopathologist experienced in prostate pathology. The histopathologist was blinded as to the genotype of the mice from where the tumor originated. The tissues sections were graded using the criteria of Gingrich et al [47]. Briefly, (*a*) normal epithelium was assigned a score of 1; (*b*) low grade prostatic intraepithelial neoplasia (PIN) with tufting of the epithelium and increased nucleus to cytoplasm ratio were scored as 2; (*c*) high grade PIN with noted cribiform structures and an increase in mitotic and/or apoptotic figures was scored as 3; (*d*) well-differentiated adenocarcinoma with the loss of interductal spaces and the invasion of basement membranes was scored as 4; (*e*) moderately-differentiated adenocarcinoma with total loss of ductal lumens with evidence of adenocarcinoma was scored as 5; and (*f*) poorly-differentiated adenocarcinoma with sheets of anaplastic cells were scored as 6. Each section from each mouse was graded and the proportion of the prostate affected by each grade was estimated. The data from all sections in a given mouse were pooled to provide an average proportion of the prostate affected by each prostate cancer grade.

### Identification of Tumor Metastases

We estimated the occurrence of metastasis at the time of death or culling in survival group TRAMP and TRAMP^fmsmic-1^ mice. At the necropsy pelvic lymph nodes, kidney, liver and rectal tumors (if present) were harvested and fixed in 10% neutral buffered formalin. Lungs were excised, weighed and fixed in Bouin’s fixative (Sigma-Aldrich) to visualize and count lung tumor colonies. Metastatic lesions on all the organs were counted under a dissecting microscope. Some of the lesions were confirmed by H&E staining and further by immunostaining of frozen tissue sections with anti Tag antibody (Santa Cruz) to confirm the prostatic origin of the tumor.

To ensure the accuracy of metastasis data obtained from the survival group mice, we examined a further cohort of TRAMP (n = 59) and TRAMP^fmsmic-1^ (n = 33) mice. In this group, we determined the proportion of mice with metastasis that died between 18–40 weeks, a period where TRAMP and TRAMP^fmsmic-1^ mice reach ethical end points at the same rate.

### Evaluation of *MIC-1/GDF15* Expression by qRT-PCR

*MIC-1/GDF15* expression in prostates of 8, 17 and 25 weeks TRAMP (n = 5) and TRAMP^fmsmic-1^ (n = 5) mice was determined by qRT-PCR and normalized to *B-actin* (house keeping gene) expression. Samples were collected and placed immediately into 1 ml of RNAlater (Qiagen) for storage at −20°C until extraction. RNA was extracted using Trizol (Invitrogen) and Ambion Purelink RNA Mini Kit (Invitrogen) with on column DNase step performed using RNA-Free DNase Set (Qiagen). AMV reverse transcriptase (Roche Applied Sciences) was used to perform reverse transcription on 2 µg of RNA from each sample. The specific primers for PCR for mouse *MIC-1/GDF15* were, Forward: 5′-AGGACTCGATCAGAACCAAG-3′ and Reverse: 5′-CGGTTGACGCGGAGTAGCA-3′ and for *B-actin* were Forward: 5′-TGACAGGATGCAGAAGGAGATTACTG-3′ and Reverse: 5′-CCACCGATCCACACAGAGTACTTG-3′. Diluted cDNA samples (1∶10) were subjected to PCR amplification with 25 mM MgCl_2_ (Roche Applied Sciences), Taq polymerase (Roche Applied Sciences) and SYBR Green I dye (Invitrogen). qRT-PCR was performed on Lightcycler LC480 instrument (Roche Applied Sciences). The reaction mixture was denatured at 95°C for 10 s, followed by 60 to 67°C annealing for 15 s, and extension at 72°C for 20 s. The specificity of the products was verified by melting curves generated by the Lightcycler 480 software version 1.5 and by PCR product size on electrophoretic gels. Gene copy numbers for both *MIC-1/GDF15* and *B-actin* were calculated by making standard curves generated by amplifying serially diluted *MIC-1/GDF15* and *B-actin* PCR products. Cp values of samples were determined using second derivative calculations performed by Lightcycler 480 software version 1.5. *MIC-1/GDF15* copy numbers were normalized to *B-actin* copy number.

Relative *MIC-1/GDF15* expression in TC1-T5 cell line was also estimated by qRT-PCR and compared with that in advanced TRAMP and TRAMP^fmsmic-1^ prostate tumors. TC1-T5 cells were grown in six well plates as below. RNA from TC1-T5 cells (n = 3) was extracted using RNeasy Mini Kit (Qiagen). RNA from TRAMP (n = 3) and TRAMP^fmsmic-1^ (n = 3) prostate tumor was extracted as described above. 2 ug RNA from each sample was reverse transcribed and subjected to qRT-PCR using the same procedure and *MIC-1/GDF15* primer set as above. *TBP* was amplified as a housekeeping gene using primer set Forward: 5′-ACCCTTCACCAATGACTCCTATG-3′ and Reverse: 5′-ATGATGACTGCAGCAAATCGC-3′. The specificity of the products was verified by melting curves generated by the Lightcycler 480 software Version 1.5 and by size on electrophoretic gels. Relative ratio of *MIC-1/GDF15* to *TBP* was then calculated using Lightcycler 480 software Version 1.5.

### Estimation of TC1-T5 TRAMP Lung Tumors

TRAMP C1 (TC1) is an androgen dependant PCa cell line derived from TRAMP prostate tumor by Foster et al. [48]. TC1-T5 is an androgen independent subline of TC1 cell line [49]. TC1-T5 cells do not express MIC-1/GDF15 in culture and are thus suitable to assess the effects of MIC-1/GDF15. TC1-T5 cells were grown in DMEM media (Invitrogen, Life Technologies) containing 5% of charcoal dextran stripped fetal-calf serum and 125U/L insulin (Humulin-BD Biosciences). To directly test whether TC1-T5 cells would metastasise more in MIC-1^fms^ mice, we injected 5×10^5^ cells in a total volume of 100 µl via the tail vein of 9-week-old MIC-1^fms^ (n = 17) and syngeneic C57BL/6 (n = 14) mice. Ten weeks later, mice were sacrificed; their lungs were excised and fixed in Bouin’s fixative (Sigma-Aldrich). Lung colonies on the surface of all the lobes of both lungs were counted under a dissecting microscope.

### Statistical Analysis

Statistical evaluations of all the experiments were performed with GraphPad Prism software version 5 (GraphPad Software, San Diego, CA, USA). All the data are presented as the mean ± standard error of the mean (SEM). Comparisons between groups were made using unpaired *t* tests or Chi-square test as appropriate and the two-tailed p values reported. Survival curves were analyzed by Kaplan–Meier analysis and log-rank statistic is reported. To examine the relationship of metastasis with survival time we used multivariate analysis. All factors significantly associated with metastasis by univariate regression were included in a multivariate regression model with survival time. A p value less than 0.05 considered statistically significant.

## Results

### Overexpression of MIC-1/GDF15 Prolongs PCa Survival in TRAMP Mice

**Figure 1:**
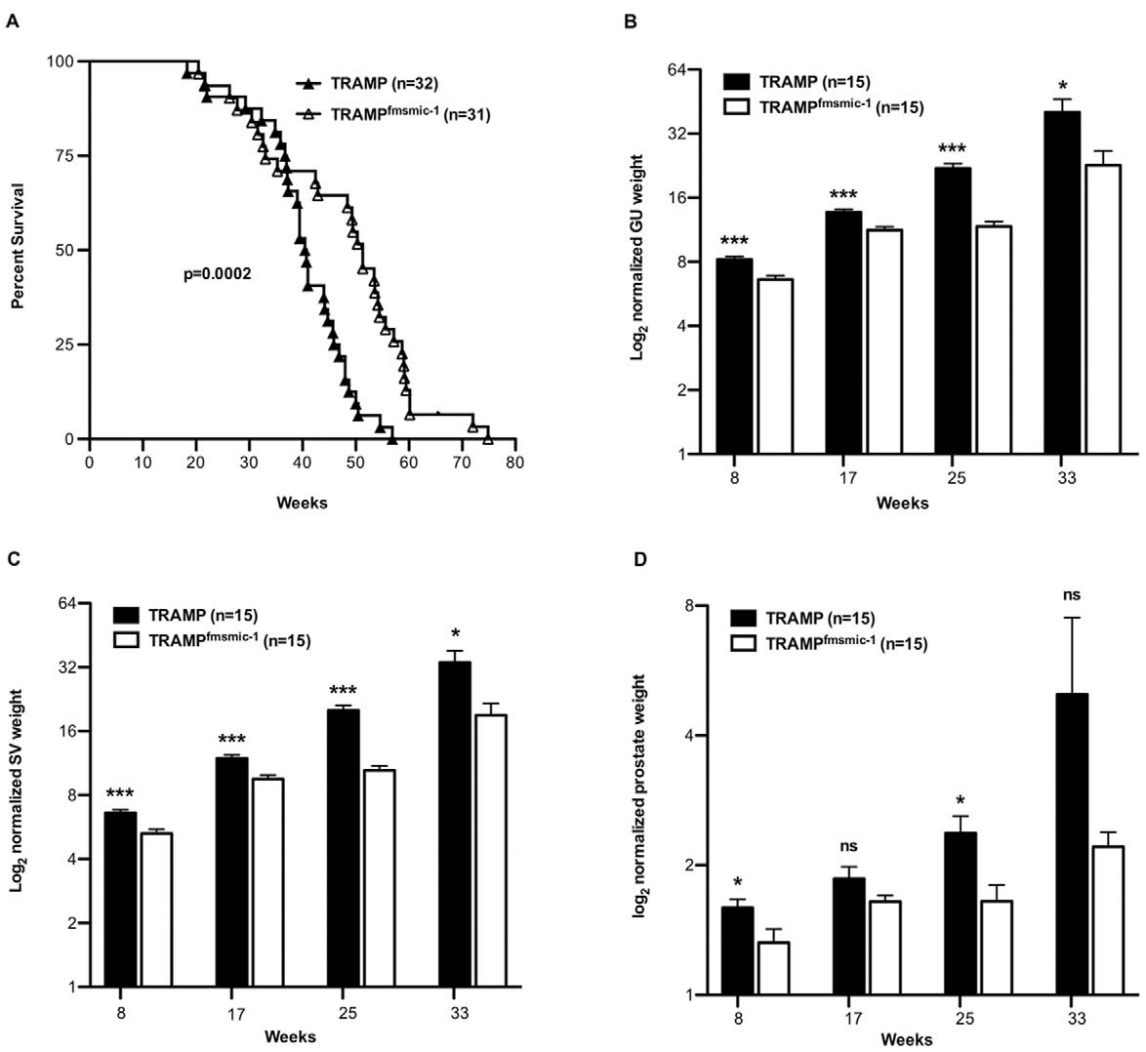
TRAMP^fmsmic-1^ mice survive longer and have smaller prostate tumors than TRAMP mice. Overall survival of individual mice from birth to death is shown (panel A). The survival data for TRAMP (—▴—) and TRAMP^fmsmic-1^ (—Δ—) mice were plotted using the Kaplan-Meier method. The log-rank statistic for median survival time is shown. The genitourinary (GU), seminal vesicle (SV) and prostate tumor sizes in TRAMP^fmsmic-1^ and TRAMP mice (n = 15/group) sacrificed at 8, 17, 25 and 33 weeks of age are shown in panel B, C and D. The GU (panel B), SV (panel C) and prostate weights (panel D) are presented as mean mg weight/g body weight ± SEM. p values are shown as *p*<*0.05; **p*<*0.01; ***p*<*0.001.

In order to assess the effects of MIC-1/GDF15 on the overall survival of TRAMP mice, we monitored a cohort of TRAMP and TRAMP^fmsmic-1^ mice till death or ethical end point. Kaplan-Meier survival analysis showed that double transgenic TRAMP^fmsmic-1^ survived significantly longer than TRAMP mice (Fig. 1A, p* = *0.0002, log-rank test).

Initially, both TRAMP and TRAMP^fmsmic-1^ mice died at a similar rate. However, after about 38-40 weeks, TRAMP mice started to succumb faster than the TRAMP^fmsmic-1^ mice. The 50% survival of 40 weeks in TRAMP mice was extended to 51 weeks in the TRAMP^fmsmic-1^ group. Further, whilst only 9.4% of TRAMP mice survived at week 50, 52% of TRAMP^fmsmic-1^ mice were still alive (Fig. 1A). Over all, the average survival time for TRAMP^fmsmic-1^ mice was 47.4 weeks and was 41 weeks for the TRAMP mice. Thus, on average, TRAMP^fmsmic-1^ mice survived approximately 7.4 weeks (∼2 months) longer than the TRAMP mice. These data indicate that increased expression of MIC-1/GDF15 is able to substantially improve PCa related survival in TRAMP mice.

### Overexpression of MIC-1/GDF15 Restrains PCa Growth

To directly assess the impact of MIC-1/GDF15 on prostate cancer growth, we assigned 60 TRAMP and 60 TRAMP^fmsmic-1^ mice at 4–6 weeks of age and culled them progressively throughout 8–33 weeks of age. Comparisons were made using the 15 mice pre-assigned to each group at the four different time points (8, 17, 25 and 33 weeks), representing very early to advanced tumor stages. Data from mice sacrificed at each time point indicated that TRAMP^fmsmic-1^ mice had significantly smaller GU complexes than TRAMP mice (Fig. 1B) at all stages (p = 0.0004, p = 0.0005, p<0.0001, and p = 0.02, respectively).

When prostate and SV weights were analyzed separately from the GU complex, SV tumor sizes were significantly reduced in TRAMP^fmsmic-1^ mice at all time points (Fig. 1C). A similar, but more varied, effect of MIC-1/GDF15 overexpression was observed on prostate tumor growth (Fig. 1D). There was 17.0% and 26% reduction in prostate tumor weight at weeks 8 and 25 respectively in TRAMP^fmsmic-1^ as compared to TRAMP mice (p* = *0.04 and 0.01, respectively). Although there was reduced tumor weight in TRAMP^fmsmic-1^ mice at 17 weeks (>10%), the difference between the two groups was not significant. At 33 weeks, although there was 55% reduction in the average prostate weight in TRAMP^fmsmic-1^ mice compared with that in TRAMP mice, the difference between the two groups was not significant because of the large variation in the prostate tumor size in the TRAMP group. Additional mice needed to be assigned to this group as many of the originally assigned mice died/reached ethical end point between 23 and 33 weeks, potentially biasing the TRAMP results, by the selection of TRAMP mice with smaller tumors. These mice with smaller tumors were more likely to survive to 33 weeks of age and be selected. However, the data suggested, consistent with the survival cohort data, that MIC-1/GDF15 overexpression decreases tumor growth.

### TRAMP^fmsmic-1^ Mice have Lower Grade Cancers

**Figure 2:**
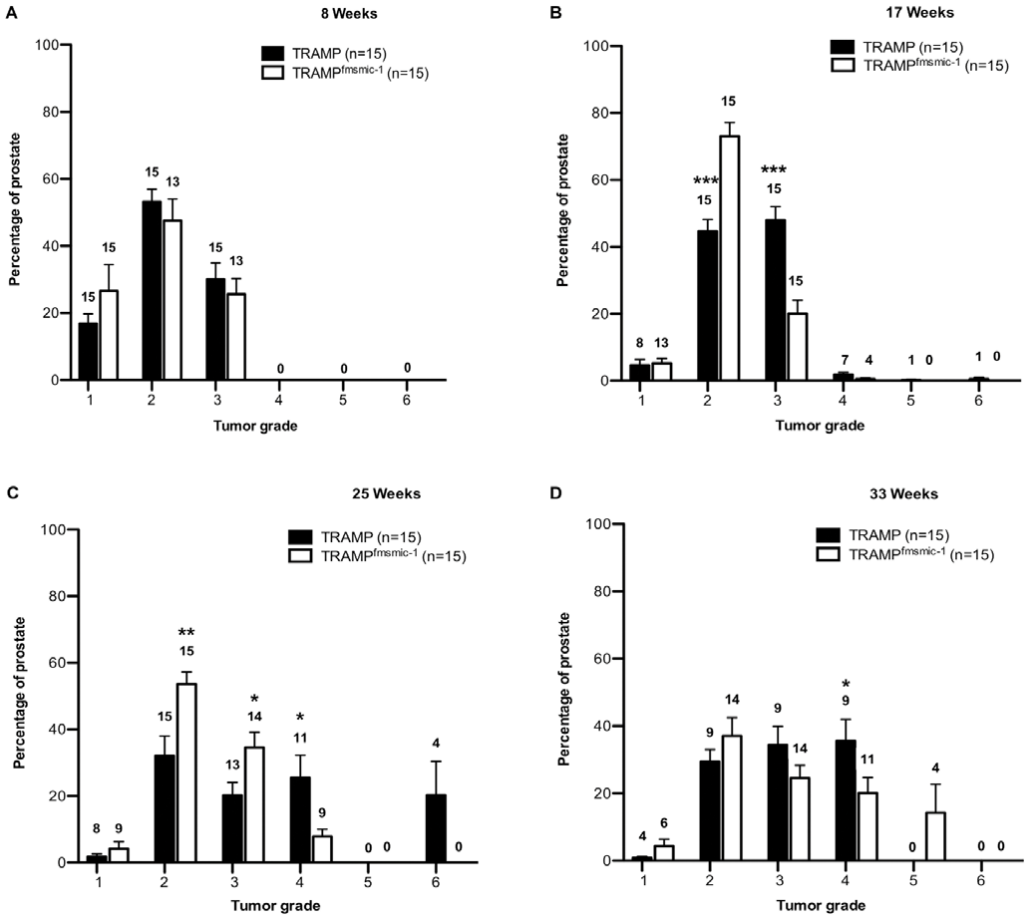
TRAMP^fmsmic-1^ prostate tumors have lower histological grades. Prostates of 15 TRAMP and 15 TRAMP^fmsmic-1^ mice were excised at week 8 (panel A), 17 (panel B), 25 (panel C), and 33 (panel D). Serial sections were graded and scores for each grade were averaged for all the mice in the group. The graph represents the mean proportion of the prostate from all the mice in the group having each different pathologic grade, scored between 1 and 6± SEM. Numbers on the bars show number of mice in the group having a particular grade. p values are shown as *p*<*0.05; **p*<*0.01; ***p*<*0.001.

In addition to following the tumor growth, we determined whether MIC-1/GDF15 might also affect PCa grade, by comparing histopathological scores in TRAMP and double transgenic TRAMP^fmsmic-1^ prostate tumors. At week 8, low (Grade 2) and high (Grade 3) grade PIN were the dominant pathologies in TRAMP and TRAMP^fmsmic-1^ mice. Whilst the average proportion of the prostate affected by PIN was higher in TRAMP compared to TRAMP^fmsmic-1^ mice; however, this difference was not statistically significant (Fig. 2A).

At week 17, Grade 2 and Grade 3 pathologies were still dominant in both of the mouse lines but Grade 4, 5 and 6 tumors started emerging in the TRAMP prostates. At this time point TRAMP mice had significantly more Grade 3 disease (p<0.0001) and significantly less Grade 2 disease (p<0.0001) than TRAMP^fmsmic-1^ mice indicating more rapid tumor progression (Fig. 2B).

At 25 weeks of age, Grade 4 tumors were more prominent in TRAMP mice but regions of Grade 2 and Grade 3 tumor were still present in both mouse lines. Comparison between individual tumor grades in the TRAMP and TRAMP^fmsmic-1^ prostate showed that a higher percentage of the TRAMP^fmsmic-1^ prostate had the lower Grades 2 and 3 tumors (p* = *0.004 and 0.02, respectively) whilst a significantly lower percentage of TRAMP^fmsmic-1^ prostate had the more aggressive Grade 4 tumors (Fig. 2C, p* = *0.019).

By 33 weeks age (Fig. 2D), most of the mice, in both groups, had advanced disease and the difference in the distribution of tumor grades between the TRAMP and TRAMP^fmsmic-1^ mice was not significant. Again these data may have been influenced by the selection bias in the 33 weeks TRAMP group, discussed above. Mice in the TRAMP group with more aggressive tumors died prior to 33 weeks of age, and only mice with the less aggressive tumors survived to this age. Overall, these data suggested that TRAMP^fmsmic-1^ mice have a larger proportion of the prostate at a lower tumor grade than TRAMP mice (Fig. 2A, B, C and D), indicating the MIC-1/GDF15 overexpression slows local tumor evolution.

### TRAMP Prostate Lacks MIC-1/GDF15 Expression

**Figure 3:**
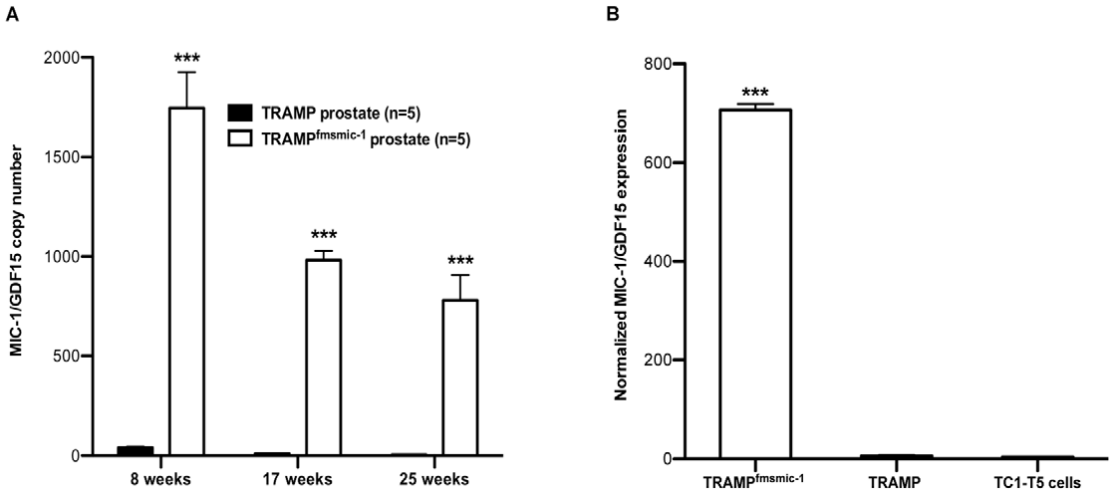
*MIC-1/GDF15* expression in TRAMP and TRAMP^fmsmic-1^ prostate and TC1-T5 cell line. (A) *MIC-1/GDF15* expression was quantified by qRT-PCR in the prostate of TRAMP and TRAMP^fmsmic-1^ mice at 8, 17 and 25 weeks and normalized to *B-actin* expression as described in material and methods. (B) Relative *MIC-1/GDF15* expression was quantified by qRT-PCR in the TC1-T5 cell line (n = 3) and compared with TRAMP (n = 3) and TRAMP^fmsmic-1^ (n = 3) prostate tumor after normalizing with *TBP* expression. Values are presented as mean normalized *MIC-1/GDF15* expression ±SEM. p values for the two-tailed unpaired *t* test are shown as ***p*<*0.001.

In order to rule out the possibility that expression of MIC-1/GDF15 by cancers in the TRAMP mice might impact upon tumor growth and modify differences between them and mice engineered to overexpress this cytokine, we quantified MIC-1/GDF15 expression in the prostate and seminal vesicle tumors from TRAMP and TRAMP^fmsmic-1^ mice. Due to unavailability of an established mouse MIC-1/GDF15 ELISA and a specific anti mouse MIC-1/GDF15 monoclonal antibody, we used qRT-PCR to compare the MIC-1/GDF15 expression in TRAMP and TRAMP^fmsmic-1^ prostate and primary tumors. There was negligible MIC-1/GDF15 expression in TRAMP mice, which are wild type for MIC-1/GDF15, at 8, 17 and 25 weeks of age (Fig. 3A) and with advanced tumors (Fig. 3B). By contrast TRAMP^fmsmic-1^ mice expressed 44–166 fold more MIC-1/GDF15 (Fig. 3A) in their prostate that is consistent with c-fms driven MIC-1/GDF15 transgene expression from infiltrating myeloid cells.

### Whilst TRAMP^fmsmic-1^ Mice Live Longer they have More Metastasis

**Figure 4:**
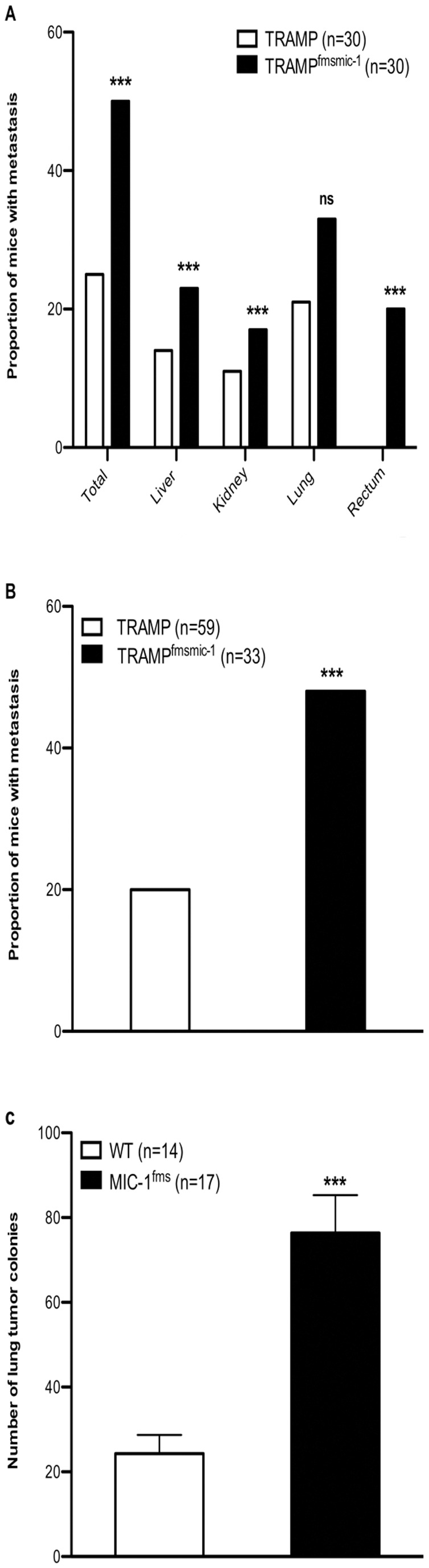
MIC-1/GDF15 increases metastasis. (A) Comparison between proportion of TRAMP (n = 30) and TRAMP^fmsmic-1^ (n = 30) mice having distant organ metastasis. The data is analyzed by Chi-square test. (B) Comparison between proportion of TRAMP (n = 59) and TRAMP^fmsmic-1^ (n = 33) mice with metastasis at 18–40 weeks, a period where TRAMP and TRAMP^fmsmic-1^ mice reach ethical end points at the same rate. The data is analyzed by Chi-square test. (C) Comparison between number of lung tumor colonies in MIC-1^fms^ mice and control C57BL/6 mice injected intravenously with TC1-T5 cells. The data is analyzed by Unpaired *t* test. The p values for Chi-square test and Unpaired *t* test are shown as ***p*<*0.001.

Since metastasis is the major cause of death from solid tumors in patients with PCa, we evaluated the effect of MIC-1/GDF15 on incidence and extent of metastasis in TRAMP mice. Although TRAMP^fmsmic-1^ mice live longer and have slower primary tumor growth (Fig. 1 A, B, C and D), a significantly higher proportion of these mice demonstrated organs with metastasis. 15 out of 30 (50%) TRAMP^fmsmic-1^ mice had distant organ metastasis compared to 6 out of 30 (20%) of TRAMP mice (Fig. 4A). A significantly higher proportion of TRAMP^fmsmic-1^ mice had macroscopically detectible metastatic lesions in the liver, kidney and rectum. Whilst the proportion of TRAMP^fmsmic-1^ mice with surface lung tumors was also increased, this fell short of statistical significance (Fig. 4A). Interestingly rectal metastases developed in 6 out of 30 TRAMP^fmsmic-1^ but not in any of the TRAMP mice (Fig. 4A). There was no difference in lymph node weight (data not shown), suggesting that lymphatic spread was not increased in TRAMP^fmsmic-1^ mice. To ensure that the increase in the number of metastasis was not solely due to increased survival times we examined the data available by multivariate logistic regression. This analysis confirmed that increase in metastasis was independent of length of survival (p = 0.03; multivariate regression). Further, it was notable that this occurred in the face of smaller tumors and lower tumor grades at all stages of disease, suggesting mechanisms independent of the effect of MIC-1/GDF15 on the primary tumor.

To further ensure the accuracy of this data we examined a further cohort of 59 TRAMP and 33 TRAMP^fmsmic-1^ mice, who died or reached ethical endpoint between the ages of 18–40 week, a time period over which both groups of mice were dying at equivalent rates. We enlisted more TRAMP than TRAMP^fmsmic-1^ mice, to compensate for the lower rate of metastases in the former. Over this period, almost 50% TRAMP^fmsmic-1^ developed metastasis, which was significantly more that the 20% observed in TRAMP mice (Fig. 4B, p<0.001, Chi-square test).

### TC1-T5 TRAMP Cells Metastasise More Frequently in MIC-1^fms^ Mice

The increased metastases observed in TRAMP^fmsmic-1^ mice might be due to various factors such as an increase in the number of tumor stem cells, increased ability of tumor cells to invade the vasculature or an enhanced capacity of blood born tumor cells to adhere to, invade and grow in the metastatic target organ. As TRAMP^fmsmic-1^ mice overexpress MIC-1/GDF15 systemically, part of its prometastatic actions may relate to effects of this cytokine on the vasculature of the target organ. To test if this might be the case, we utilised the TRAMP TC1-T5 tumor cell line. These cells, which are also of C57BL/6 mouse origin and express no MIC-1/GDF15 (Fig. 3B), were injected intravenously into MIC-1/GDF15 overexpressing MIC-1^fms^ and syngeneic C57BL/6 mice. Ten weeks later, TC1-T5 lung tumor colonies were counted under a dissecting microscope. At an average of 76.35±8.931 colonies, MIC-1^fms^ mouse lungs, had almost 3 times as many tumor colonies as control mice (24.29±4.417 colonies, Fig. 4C, p<0.0001). These data indicate that at least some of the actions mediating MIC-1/GDF15 associated metastases are independent of its actions on primary tumor cells themselves and are likely to involve mechanisms such as increased tumor vascular adhesion, survival or extravasation.

## Discussion

This study relies on the use of the TRAMP PCa prone mouse. In this spontaneous transgenic mouse model, the probasin (rPB) regulatory sequence targets expression of SV40 early genes (T and t; Tag) specifically to prostatic epithelium [44]. These mice reproducibly develop PIN lesions with the onset of puberty, which then progress in a very similar manner to human PCa, making it an ideal model of early tumor development [47]. However, unlike most human PCa, TRAMP PCas do not express MIC-1/GDF15, perhaps due to the inhibition of p53, a major driver of MIC-1/GDF15 expression, by the SV40 T antigen in the TRAMP transgene [50]–[52]. Because of this, there is no background expression of MIC-1/GDF15 in the TRAMP mouse seminal vesicles or prostate to complicate the interpretation of local prostate tumor development in TRAMP^fmsmic-1^ mice. In the MIC-1^fms^ mice that contributed the MIC-1/GDF15 transgene, constitutive MIC-1/GDF15 overexpression occurs from myeloid cells [33]. Thus, whilst not directly examined as part of this study, the source of MIC-1/GDF15 overexpression in the prostates of TRAMP^fmsmic-1^ mice would be expected to be the infiltrating or tissue derived macrophages or dendritic cells known to be present in prostate cancers. Additionally, this overexpression should raise serum MIC-1/GDF15 levels in TRAMP^fmsmic-1^ mice as it does in MIC-1^fms^ mice [53]. Thus MIC-1/GDF15 could act both systemically and/or directly on the prostate epithelial cells. This is analogous to what would frequently occur in man. In human PCa tumors, most MIC-1/GDF15 is expressed by malignant epithelial cells [5], [10] although there is also be a contribution from tumor stromal cells [11] and infiltrating phagocytes [12]. Additionally, all normal individuals have circulating levels of MIC-1/GDF15 over a broad normal adult range of 150 to 1150 pg/ml [13]. These levels rise further, with aging, intercurrent diseases, pregnancy and in cancers. Some premalignant lesions such as colonic adenomas, can also further increase serum MIC-1/GDF15 levels [13].

There is a dearth of information about the effects of MIC-1/GDF15 in spontaneous models of cancer development, which more closely correspond to cancer biology in man. For MIC-1/GDF15, the only other study in transgenic cancer models, have been in the APC^min/+^ small and large bowel polyp prone mice. Consistent with our current data, mice systemically overexpressing MIC-1/GDF15 were relatively protected from developing small bowel polyps [54]. Additionally, MIC-1/GDF15 gene deleted, APC^min/+^ mice loose non-steroidal anti-inflammatory drug (NSAID) induced chemoprevention of colonic neoplasia [55]. The latter findings are also reflected in human studies as NSAIDs use leads to a rise in serum MIC-1/GDF15 levels that is associated with protection from colonic polyposis [56].

Whilst MIC-1/GDF15 has a favorable impact on the growth of primary tumors, its overexpression causes a major increase on the proportion of mice with metastases. Whilst slower primary tumor growth and more metastases, might seem counterintuitive, this can occur because many aspects of metastasis are regulated independently of tumor growth. This apparently paradoxical action is also not unprecedented and is displayed by TGF-β, a distant relative of MIC-1/GDF15 [57].

At least some of the causes of increased metastases in TRAMP^fmsmic-1^ mice must be independent of any MIC-1/GDF15 induced alteration to the primary tumor. The TC1-T5 TRAMP cell line, that makes no MIC-1/GDF15, when injected intravenously, is far more metastatic into transgenic MIC1/GDF15 overexpressing MIC-1^fms^ mice than into syngeneic WT mice. These results suggest actions on tumor cell adherence, survival or invasion.

Overall, our results support an important role for MIC-1/GDF15 in the regulation of growth and development of cancer in the TRAMP PCa prone mice. TRAMP^fmsmic-1^ mice had a significantly reduced tumor grades and primary tumor size. As a major cause of death in the C57BL/6 TRAMP model is growth and local invasion from the primary tumor, TRAMP^fmsmic-1^ mice lived significantly longer, than control TRAMP mice. However, independently of this increased survival, more TRAMP^fmsmic-1^ than TRAMP mice had metastatic deposits, suggesting that with advanced tumors, MIC-1/GDF15 may facilitate haematogenous spread. This view was further supported by our studies in which intravenous TC1-T5 TRAMP cell line injections lead to significantly more metastasis in MIC-1/GDF15 overexpressing MIC-1^fms^ mice compared with syngeneic control mice. MIC-1/GDF15 facilitated metastasis formation, if applicable to human PCa, may have implications for the cancer management, because increase in MIC-1/GDF15 expression or serum levels may impact on metastasis formation. MIC-1/GDF15 serum levels may rise because of the physiological factors such as increasing age or pregnancy, production by the cancer, cancer treatment, injury or intercurrent diseases [4]. Most cancer therapeutic modalities including surgery, radiotherapy and chemotherapy [6], [7], [51] may induce MIC-1/GDF15 expression, which can be seen both in cancer and non-cancer tissues. Thus it is possible that in addition to its beneficial effects, anticancer therapy may sow the seeds of metastatic recurrence.
